# Latent Transforming Growth Factor-β Binding Protein-2 Regulates Lung Fibroblast-to-Myofibroblast Differentiation in Pulmonary Fibrosis *via* NF-κB Signaling

**DOI:** 10.3389/fphar.2021.788714

**Published:** 2021-12-24

**Authors:** Menglin Zou, Jingfeng Zou, Xingxing Hu, Weishuai Zheng, Mingyang Zhang, Zhenshun Cheng

**Affiliations:** ^1^ Department of Respiratory and Critical Care Medicine, Zhongnan Hospital of Wuhan University, Wuhan, China; ^2^ Department of Respiratory and Critical Care Medicine, Jiangxi Provincial People’s Hospital, Nanchang, China

**Keywords:** pulmonary fibrosis, latent transforming growth factor-β binding protein-2, fibroblast-to-myofibroblast differentiation, extracellular matrix, NF-κB signaling

## Abstract

Despite past extensive studies, the mechanisms underlying pulmonary fibrosis (PF) still remain poorly understood. The aberrantly activated lung myofibroblasts, predominantly emerging through fibroblast-to-myofibroblast differentiation, are considered to be the key cells in PF, resulting in excessive accumulation of extracellular matrix (ECM). Latent transforming growth factor-β (TGFβ) binding protein-2 (LTBP2) has been suggested as playing a critical role in modulating the structural integrity of the ECM. However, its function in PF remains unclear. Here, we demonstrated that lungs originating from different types of patients with PF, including idiopathic PF and rheumatoid arthritis-associated interstitial lung disease, and from mice following bleomycin (BLM)-induced PF were characterized by increased LTBP2 expression in activated lung fibroblasts/myofibroblasts. Moreover, serum LTBP2 was also elevated in patients with COVID-19-related PF. LTBP2 silencing by lentiviral shRNA transfection protected against BLM-induced PF and suppressed fibroblast-to-myofibroblast differentiation *in vivo* and *in vitro*. More importantly, LTBP2 overexpression was able to induce differentiation of lung fibroblasts to myofibroblasts *in vitro*, even in the absence of TGFβ1. By further mechanistic analysis, we demonstrated that LTBP2 silencing prevented fibroblast-to-myofibroblast differentiation and subsequent PF by suppressing the phosphorylation and nuclear translocation of NF-κB signaling. LTBP2 overexpression-induced fibroblast-to-myofibroblast differentiation depended on the activation of NF-κB signaling *in vitro*. Therefore, our data indicate that intervention to silence LTBP2 may represent a promising therapy for PF.

## Introduction

Pulmonary fibrosis (PF) is a type of chronic and progressive lung interstitial disease, characterized by the irreversible scarring and remodeling of the lung (Kolahian et al., 2016). The fibrogenic triggers remain controversial, but identifiable triggers probably include cigarette smoke (Wilson and Wynn, 2009), respiratory infections such as the 2019 novel coronavirus disease (COVID-19) (George et al., 2020), and connective tissue diseases/autoimmune disorders such as rheumatoid arthritis (RA) and scleroderma (Wilson and Wynn, 2009; Spagnolo et al., 2018). However, PF can also manifest without any known etiology, which is termed “idiopathic” (IPF) (Lederer and Martinez, 2018). To date, the mechanisms underlying PF remain poorly understood, so there are limited therapeutic options and poor prognosis. Therefore, novel agents targeted against the fibrotic process are urgently needed.

The currently accepted pathogenic theories suggest that formation of fibrotic foci and excessive deposition of extracellular matrix (ECM) proteins, such as hyaluronan, fibronectin, and interstitial collagens, are principal events in PF (Kolahian et al., 2016). Following lung injury, quiescent fibroblasts become activated and transform into α-smooth muscle actin (α-SMA)-expressing myofibroblasts, resulting in excess accumulation of ECM components (Wynn, 2011). Thus, targeting the fibroblast-to-myofibroblast differentiation process and ECM synthesis would provide a potential therapeutic strategy for PF.

Latent transforming growth factor-β (TGFβ) binding protein-2 (LTBP2) is a member of the fibrillin/LTBP ECM proteins family, which includes LTBP-1, -2, -3, and -4 (Gibson et al., 1995; Robertson et al., 2015). LTBPs play a critical role in modulating the structural integrity of the ECM, and in the assembly and secretion of the latent TGF-β (Miyazono et al., 1991). Unlike the other LTBPs, LTBP2 is unable to bind to latent TGFβ (Anderson et al., 2008; Robertson et al., 2015). Thus, the function of LTBP2 has not been fully clarified. Previous studies have demonstrated that high expression of LTBP2 was associated with poor outcome and tumor progression in thyroid and gastric cancer. Moreover, knockdown of LTBP2 inhibited the proliferation and invasion in thyroid carcinoma and gastric cancer cells (Wan et al., 2017; Wang et al., 2018). Enomoto et al. (2018) found that LTBP2 was secreted from lung myofibroblasts and was potentially a novel prognostic blood biomarker reflecting the level of differentiation of lung fibroblasts into myofibroblasts in IPF patients. However, there has been very little research using LTBP2-knockout mice to assess lung fibrosis, and the exact function of LTBP2 in lung (myo) fibroblasts and PF remains unknown.

Nuclear factor kappa B (NF‐κB), a well-characterized transcription factor, regulates many genes responsible for the generation of proinflammatory molecules and profibrogenic cytokines, which promote inflammatory response and fibrosis (Christman et al., 2000; Wilson and Wynn, 2009; Meng et al., 2014). The activation of the NF-κB signaling pathway resulted in the apoptotic resistance of lung fibroblasts (Golan-Gerstl et al., 2012), but blocking NF-κB signaling attenuated bleomycin (BLM)-induced lung fibrosis *via* suppressing myofibroblast differentiation (Hou et al., 2018). A previous study showed that LTBP2 knockdown by siRNA reduced the degree of myocardial fibrosis by suppressing activation of the NF-κB signaling pathway (Pang et al., 2020). However, research regarding whether the NF‐κB signaling is related to LTBP2 in PF has rarely been reported.

In the current study, we characterized the expression pattern of LTBP2 and investigated for the first time its role in lung fibroblasts’ differentiation to myofibroblasts and lung fibrosis. We showed that LTBP2 silencing by lentiviral shRNA transfection attenuated PF by suppressing lung fibroblast-to-myofibroblast differentiation and ECM deposition *via* blocking NF-κB signaling.

## Materials and Methods

### Human Samples

Lung tissues from patients with IPF (n = 3) and rheumatoid arthritis-associated interstitial lung disease (RA-ILD) (n = 3) were collected in the Zhongnan Hospital of Wuhan University. Three normal lung biopsies from resection of cancer were used as controls. IPF patients fulfilled the American Thoracic Society (ATS)/European Respiratory Society (ERS) consensus diagnostic criteria (Raghu et al., 2018). RA was classified according to the 1987 American College of Rheumatology (ACR) classification criteria (Arnett et al., 1988) and a definitive diagnosis of ILD was evident by clinical features, chest high-resolution computed tomography (HRCT), laboratory findings, and/or surgical lung biopsy. The pattern in RA-ILD patients was usual interstitial pneumonia (UIP) based on HRCT and/or histopathological pattern in this study. COVID-19 diagnosis was made according to the nucleic acid test, and PF in COVID-19 survivors was diagnosed by chest CT images. Six patients with COVID-19-related PF and 13 age-, and male sex-matched COVID-19 survivors without PF were included in the present study. As healthy controls, serum samples were also collected from 11 healthy volunteers who worked or had worked at Zhongnan Hospital of Wuhan University.

### BLM-Induced Lung Fibrosis Model

We used 8- to 10-week-old male C57BL/6 mice housed in a specific pathogen-free environment with sterilized food and water. For BLM-induced fibrosis studies, mice under pentobarbital anesthesia received a single intratracheal injection of 3 mg/kg BLM (Hisun, Zhejiang, China) dissolved in 50 μl of saline. Control mice were intratracheally injected with 50 μl saline. At 28 days later, mice were euthanized. Parts of the lung lobes were fixed in 4% paraformaldehyde (PFA) for histopathologic analyses, and parts were frozen for further analysis.

### Lentivirus Production and Transfection

Three different mouse LTBP2 shRNAs and scrambled shRNA were designed and synthesized by GeneChem (Shanghai, P.R. China). The target sequences of shRNAs are shown in Supplementary Table S1. The shRNA1 with the best silencing efficiency was chosen for further animal experiments. C57BL/6 mice under anesthesia were intratracheally administrated with scrambled shRNA or LTBP2 shRNA at a dose of 3.5 × 10^7^ transduction units per mouse. Five days later, mice were challenged with BLM for lung fibrosis experiments.

### Lung Histological Analysis

The lower lung tissues were fixed in 4% PFA for 24 h, sliced mid-sagittally, and embedded in paraffin. Then 4 μm sections were stained with hematoxylin-eosin (H&E) for structured observation, or with Masson’s trichrome staining for detection of collagen deposition. In H&E staining, the severity of fibrosis was estimated from 0 to 8 by the Ashcroft score (Ashcroft et al., 1988).

### Immunohistochemistry Staining

Tissue sections were deparaffinized and heated in EDTA buffer, followed by treatment with 3% H_2_O_2_ in methanol for 10 min and blocking with 5% normal serum. Tissue sections were incubated with primary antibody overnight at 4°C, and then incubated with secondary antibody for 50 min at room temperature, visualized with DAB chromogen, and counterstained with hematoxylin.

### FACS Protocol

Mouse lung cells from the BLM 28 days group were incubated with antibodies against the following lineage-specific cell surface markers: CD45, CD31, viability dye, CD11b, and CD11c. When isolating lung fibroblasts from mice, anti-PDGFRα antibody was added. We defined fibroblasts as lineage-negative and PDGFRα-positive cells. Then we further analyzed the transcription of key profibrotic genes in LTBP2-expressing and LTBP2-negative lung fibroblasts.

### Hydroxyproline Assay

Total hydroxyproline content of lung tissues were analyzed with the Hydroxyproline Assay Kit (Cat. No: A030-2, Jiancheng, Nanjing, China) according to the manufacturer’s protocol. In brief, the lung tissue homogenates were centrifuged and the supernatant was collected. Subsequently, hydroxyproline content in lung tissues was evaluated by the absorbance at 550 nm. The results were expressed as μg hydroxyproline/mg wet lung.

### Culture and Lentiviral Infection of HFL1 Cells

HFL1 cells, the human pulmonary fibroblast cell line, were purchased from iCell Bioscience (Shanghai, China). HFL1 cells were cultured in complete medium containing Kaighn’s modification of Ham’s F-12 (F12K) medium (Hyclone, Logan, UT, United States) supplemented with 10% fetal bovine serum (FBS, Gibco; Thermo Fisher Scientific, Inc., Waltham, MA, United States) and 1% antibiotics (100 μg/ml streptomycin and 100μ/ml penicillin) in a humidified atmosphere of 5% CO_2_ at 37°C. Three different human LTBP2 shRNAs and scrambled shRNA were designed and synthesized by GeneChem (Shanghai, P.R. China). The target sequences of shRNAs are shown in Supplementary Table S2. The shRNA3 with the best silencing efficiency was chosen for further experiments. For functional studies, HFL1 cells were plated in 6-well plates (1 ×10^5^ cells/well), and infected with scrambled shRNA or LTBP2 shRNA at the multiplicity of infection (MOI = 40). After 8 h, the medium was replaced with fresh complete medium and the cells were incubated with 4 μg/ml puromycin for 72 h when the cells reached 70–80% confluence. Then the cells were stimulated with PBS or 10 ng/ml TGF-β1 (PeproTech, Rocky Hill, NJ, United States) for 48 h.

### LTBP2 Plasmids Construction and Transfection

Human LTBP2 cDNA was synthesized and cloned into GV230 vector (GeneChem, Shanghai, P.R. China). Transfection was carried out using the Lipo8000™ Transfection Reagent (Beyotime, Shanghai, China) according to the manufacturer’s instructions. HFL1 cells were pretreated with BAY 11-7082 (MedChem Express, NJ, United States) for 1 h followed by transfection with LTBP2 plasmids.

### Immunofluorescence

HFL1 cells or tissue sections were fixed in 4% PFA, then permeabilized with 0.2% Triton X-100 for 10 min, and blocked in 5% bovine serum albumin for 60 min. The cells or tissue sections were incubated with primary antibody overnight at 4°C, and subsequently incubated with appropriate secondary antibody for 1 h at room temperature. Nuclei were stained with DAPI for 3 min.

### Quantitative RT-PCR

Total RNAs were extracted from tissues and cells using TRIzol reagent (Invitrogen, Carlsbad, CA, United States). Total RNAs were reversely transcribed to cDNAs using the Prime Script RT Reagent Kit (TaKaRa, Dalian, China) according to the manufacturer’s instructions. For quantitative analysis of mRNA expression, the SYBR Green PCR kit (Toyobo, Osaka, Japan) was used to amplify the target gene using the manufacturer’s protocol. Relative mRNA levels of the target gene were normalized to GAPDH mRNA expression by using the 2^−ΔΔCT^ method.

### The Mouse Primer Sequences Used in the Study Were Listed as Follows

LTBP2: forward, 5′-ACA​CTT​GCG​ACT​GCT​TTG​AGG-3′, reverse, 5′-CAG​TGG​CAG​CGA​TAG​GAA​CC-3`; α-SMA: forward, 5′-GAT​AGA​ACA​CGG​CAT​CAT​CAC​C-3′, reverse, 5′-CAT​AAT​CTG​GGT​CAT​TTT​CTC​CC-3`; Fibronectin: forward, 5′-GCA​AGG​AAA​CAA​GCA​AAT​GC-3′, reverse, 5′-GTT​GTA​GGT​GAA​CGG​GAG​GAC-3`; Col1α1: forward, 5′-CTG​ACT​GGA​AGA​GCG​GAG​AG-3′, reverse, 5′-CGG​CTG​AGT​AGG​GAA​CAC​AC-3`; Col2α1: forward, 5′-GAC​GCC​ATG​AAA​GTT​TTC​TGC-3′, reverse, 5′-CCC​TCA​GTG​GAC​AGT​AGA​CGG-3`; IL-1β: forward, 5′-GAA​ATG​CCA​CCT​TTT​GAC​AGT​G-3′, reverse, 5′-TGG​ATG​CTC​TCA​TCA​GGA​CAG-3`; IL-6: forward, 5′-CTG​CAA​GAG​ACT​TCC​ATC​CAG-3′, reverse, 5′-AGT​GGT​ATA​GAC​AGG​TCT​GTT​GG-3`; TNFα: forward, 5′-TTC​TCA​TTC​CTG​CTT​GTG​G-3′, reverse, 5′-ACT​TGG​TGG​TTT​GCT​ACG-3`; GAPDH: forward, 5′-TGA​AGG​GTG​GAG​CCA​AAA​G-3′, reverse, 5′-AGT​CTT​CTG​GGT​GGC​AGT​GAT-3`.

### The Human Primer Sequences Used in the Study Were Listed as Follows

LTBP2: forward, 5′-ACA​GCA​AAC​AGC​ACC​AAC​CAC-3′, reverse, 5′-CTC​ATC​GGG​AAT​GAC​CTC​CTC-3`; α-SMA: forward, 5′-CTT​GAG​AAG​AGT​TAC​GAG​TTG​C-3′, reverse, 5′-GAT​GCT​GTT​GTA​GGT​GGT​TTC-3`; Fibronectin: forward, 5′-GTG​GCA​GAA​GGA​ATA​TCT​CGG-3′, reverse, 5′-TCT​AAA​GGC​ATG​AAG​CAC​TCA​A-3`; Col1α1: forward, 5′-GCC​AAG​ACG​AAG​ACA​TCC​CA-3′, reverse, 5′-CGT​CAT​CGC​ACA​ACA​CCT​TG-3`; GAPDH: forward, 5′-CAT​CAT​CCC​TGC​CTC​TAC​TGG-3′, reverse, 5′-GTG​GGT​GTC​GCT​GTT​GAA​GTC-3`.

### Western Blot Analysis

The protein concentration was measured using bicinchoninic acid assay kit (Aspen, Wuhan, China). Nuclear extracts were prepared with a nuclear and cytoplasmic protein extraction kit (Beyotime, Shanghai, China) according to the manufacturer’s instructions for the detection of NF-κB p65 and phospho-NF-κB p65 (phospho s536) protein expression. Protein samples were separated by SDS-PAGE and transferred to PVDF membranes (Millipore, Bedford, MA, United States). The membranes were then blocked with 5% non-fat milk in TBST buffer for 2 h, and subsequently incubated with the appropriate primary antibodies at appropriate dilutions overnight at 4°C. After washing, the membranes were incubated with horseradish peroxidase (HRP)-conjugated secondary antibodies at room temperature for 2 h. The bands were visualized using the electrochemiluminescence (ECL) system (Tanon, Shanghai, China). The primary antibody against antibodies to α-SMA, collagen I, fibronectin, and Glyceraldehyde-3-phosphate dehydrogenase (GAPDH) were purchased from Abcam (Cambridge, MA, United States). Antibodies for total NF-κB p65, phospho-NF-κB p65 (phospho s536) and Lamin B were purchased from Cell Signaling Technology (Danvers, MA). Antibody against LTBP2 was purchased from Santa Cruz Biotechnology (Santa Cruz, CA, United States). Goat anti-rabbit and anti-mouse horseradish peroxidase (HRP)-conjugated secondary antibodies were acquired from Aspen (Wuhan, China).

### Enzyme-Linked Immunosorbent Assay

LTBP2 protein levels in serum, bronchoalveolar lavage fluid (BALF), and cell culture media were detected using the Mouse LTBP2 ELISA Kit (ELK Biotechnology, Wuhan, China) and the Human LTBP2 ELISA Kit (ELK Biotechnology, Wuhan, China) following the manufacturers’ instructions.

### Statistical Analysis

All data were analyzed using the Statistical Package for the Social Sciences software (SPSS 21.0, Chicago, IL, United States) and presented as mean ± standard deviation (SD). Significant differences were determined by one-way ANOVA for multiple comparisons and Student’s *t*-test for pairwise comparisons between two groups. *p* values less than 0.05 were considered significant.

## Results

### LTBP2 Is Overexpressed in IPF Patients and BLM-Injured Mice

**FIGURE 1:**
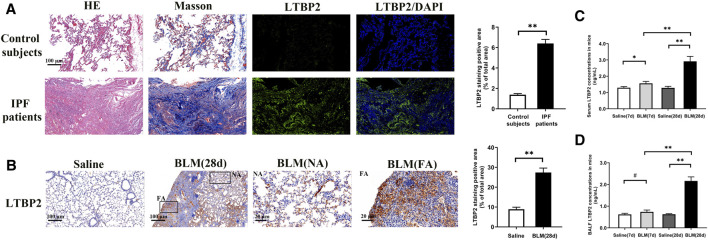
LTBP2 is overexpressed in IPF patients and mice with BLM induction. **(A)** Representative Hematoxylin-eosin staining, Masson’s trichrome staining, and immunofluorescence staining of LTBP2 (green) in the lung sections from normal people and IPF patients. Nuclei are stained with DAPI (blue); Scale bar = 100 μm. Quantification of positive staining area was measured and statistically analyzed. n = 3, **p < 0.01. **(B)** Immunohistochemistry for LTBP2 expression in the lungs of mice. Scale bar = 100 μm. NA stands for normal area; FA stands for fibrotic area; Scale bar = 20 μm. Quantification of positive staining area was measured and statistically analyzed. n = 3, **p < 0.01 **(C)** LTBP2 levels in serum of mice. n = 6, *p < 0.05, **p < 0.01 **(D)** LTBP2 levels in BALF of mice. n = 6, ^#^ *p* > 0.05, **p < 0.01. Data are represented as means ± SD. Statistical analysis was performed by one-way ANOVA or *t*-test.

To verify LTBP2 as a candidate gene in PF, we first assessed LTBP2 expression in lung tissues of IPF patients. LTBP2 was almost undetectable in normal lung tissues, but it was highly expressed in the lungs from patients with IPF (Figure 1A). To address the above assumption, we further investigated LTBP2 expression in the PF murine model with a single intratracheal administration of BLM. We also observed considerably increased LTBP2 immunohistochemical staining in active fibrotic areas (Figure 1B). Furthermore, BLM upregulated LTBP2 levels in serum and BALF of mice with PF, and LTBP2 levels in BLM 28 days group were higher than those in BLM 7 days group (Figures 1C,D). Collectively, these findings support that IPF patients and BLM-induced mice are characterized by LTBP2 overexpression.

### LTBP2 Is Co-localized in Activated Lung Fibroblasts/Myofibroblasts

**FIGURE 2:**
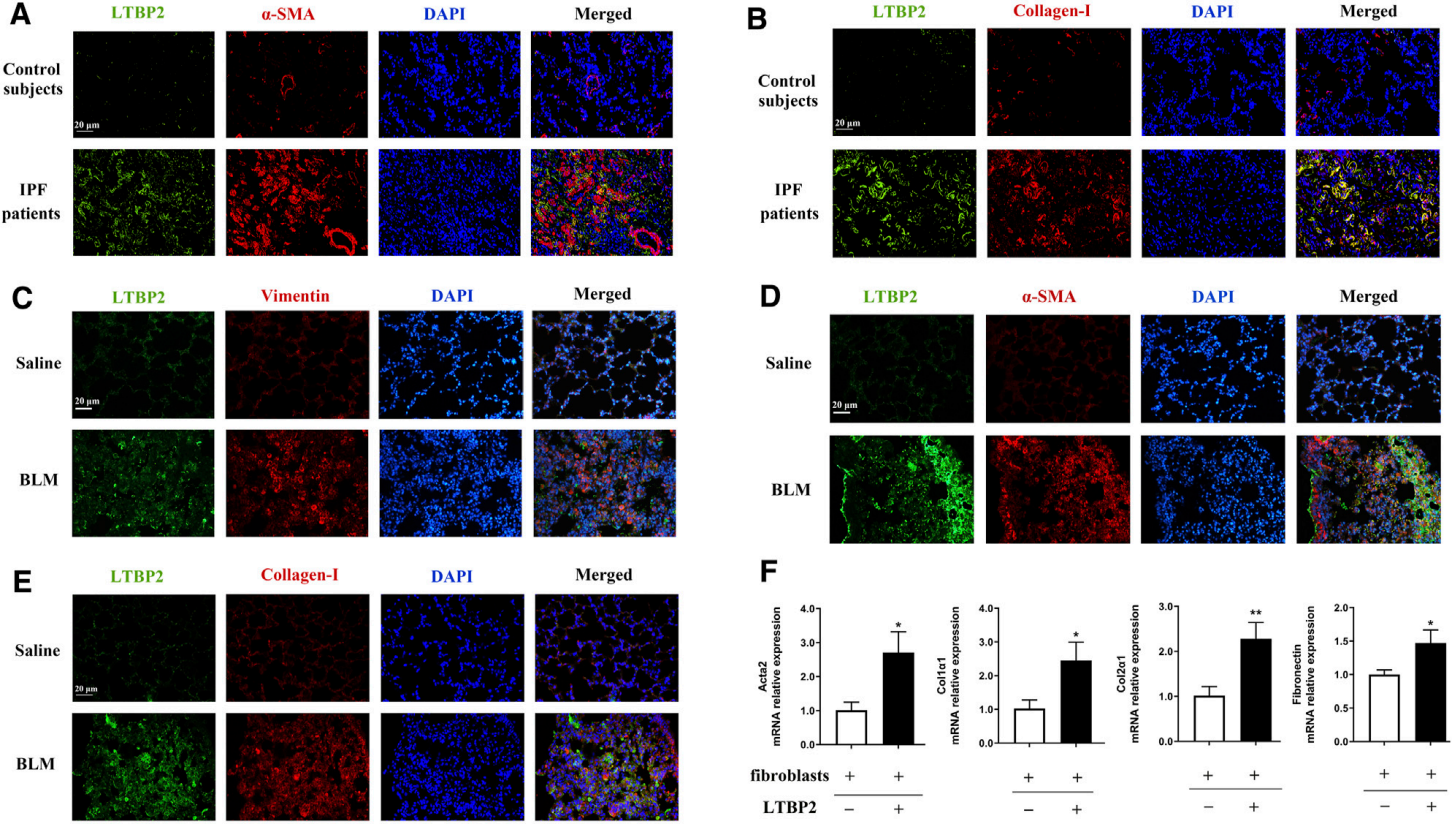
LTBP2 is co-localized in activated lung fibroblasts/myofibroblasts. **(A)** Representative results for co-immunostaining of LTBP2 (green) and α-SMA (red) in the lung sections from patients with IPF (n = 3). Scale bar = 20 μm. **(B)** Representative results for co-immunostaining of LTBP2 (green) and collagen-I (red) in the lung sections from patients with IPF (n = 3). Scale bar = 20 μm. **(C)** Results for co-immunostaining of LTBP2 (green) and vimentin (red) in mice lungs (n = 3). Scale bar = 20 μm. **(D)** Representative results for co-immunostaining of LTBP2 (green) and α-SMA (red) in mice lungs (n = 3). Scale bar = 20 μm. **(E)** Results for co-immunostaining of LTBP2 (green) and collagen-I (red) in mice lungs (n = 3). Scale bar = 20 μm. **(F)** qRT-PCR analysis of Acta2, Col1α1, Col2α1, and Fibronectin in LTBP2-expressing and LTBP2-negative lung fibroblasts (n = 3 per group). *p < 0.05, **p < 0.01. Nuclei are stained with DAPI (blue). Data are represented as means ± SD. Statistical significance was analyzed using *t*-test.

Next, we stained LTBP2 and the marker proteins of lung fibroblast activation in fibrotic lungs with IPF patients and mice. The increased expression of LTBP2 was detected in α-SMA -positive or collagen-I-positive cells, suggesting that LTBP2 was co-localized in activated fibroblasts/myofibroblasts of IPF lungs (Figures 2A,B). Murine models of PF showed that LTBP2 expression was detected in vimentin-positive fibroblasts, and the increased expression of LTBP2 was co-localized with α-SMA and collagen-I in lung fibrotic regions (Figures 2C–E). To further investigate the role of LTBP2 in lung fibroblast activation, we sorted LTBP2-expressing and LTBP2-negative fibroblasts from BLM-induced mice lungs (Supplementary Figure S1A) and analyzed the transcription of key profibrotic genes. As shown in Figure 2F, LTBP2-expressing fibroblasts expressed higher mRNA levels for Acta2, Col1α1, Col2α1, and fibronectin compared to LTBP2-negative fibroblasts. Taken together, our data indicate that LTBP2 is highly expressed in activated lung fibroblasts/myofibroblasts and positively related with lung fibroblast activation.

### LTBP2 Silencing Attenuates BLM-Induced PF in Mice

**FIGURE 3:**
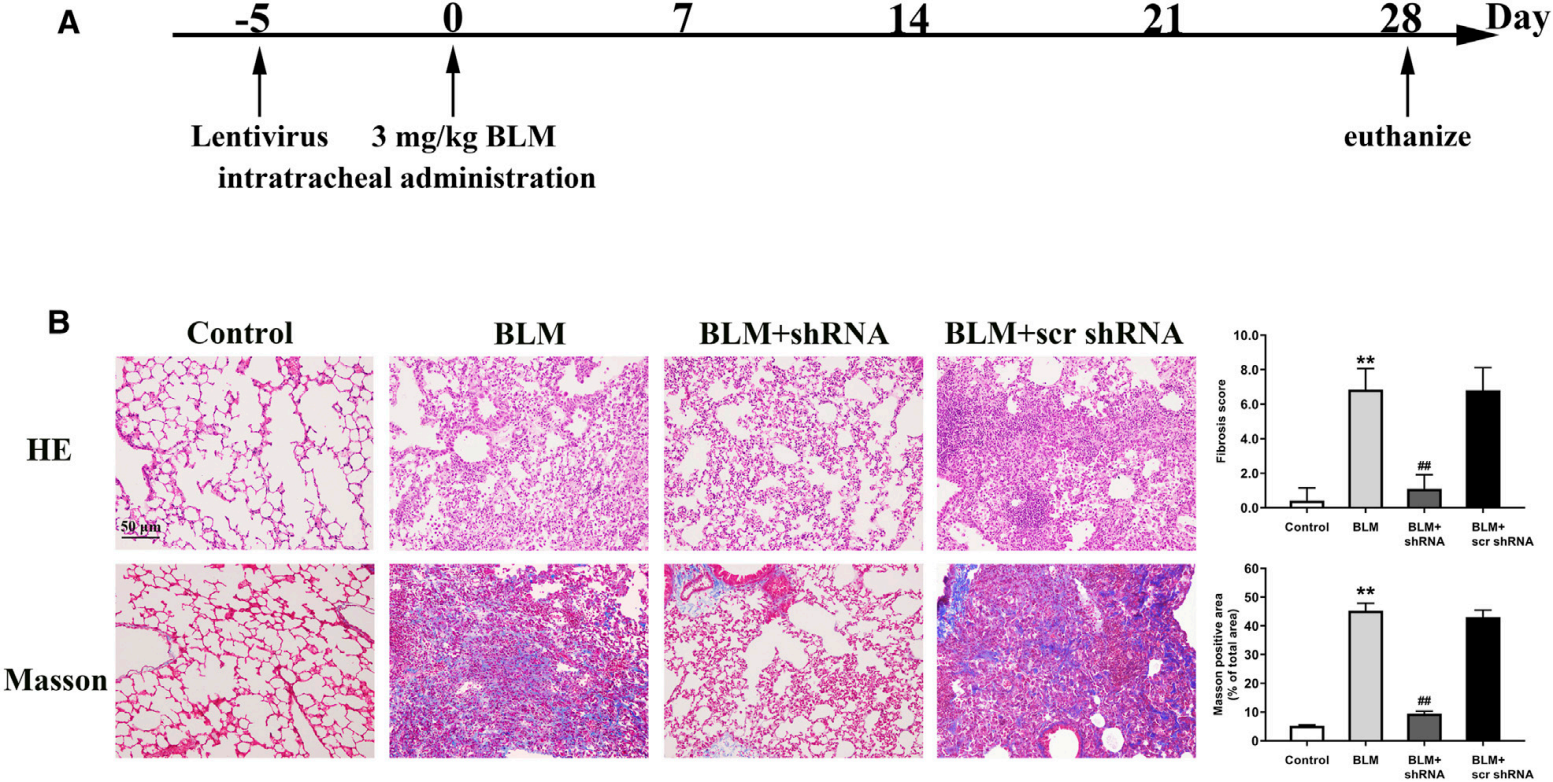
LTBP2 silencing attenuates BLM-induced pulmonary fibrosis in mice. **(A)** Strategy for knockdown LTBP2 in BLM-induced lung fibrosis mouse model. Mice were received an intratracheal injection of LTBP2 shRNA, scrambled shRNA (scr shRNA), or left untreated. Five days later, mice were received a single intratracheal administration of BLM or saline **(B)** Representative histopathological sections of lung tissues by Hematoxylin-eosin staining and Masson’s trichrome staining in mice. Scale bar = 50 μm. Fibrotic score was measured using the Ashcroft method. Quantification of positive Masson’s staining area was measured and statistically analyzed. n = 6, **p < 0.01 vs. control, ^##^ *p* < 0.01 vs. BLM + scr shRNA. Data are represented as means ± SD. Statistical analysis was performed by one-way ANOVA.

To reveal the role of LTBP2 in PF, we designed three lentivirus shRNAs for LTBP2 silencing. We chose shRNA1 for further *in vivo* experiments because of its silencing efficiency (Supplementary Figures S1B, S1C). BLM-induced increase of LTBP2 protein was dramatically reduced in mice lungs after LTBP2 shRNA administration (∼59% decrease; Supplementary Figure S1D). LTBP2 silencing attenuated the extent and intensity of the lung injury, fibrosis regions formation, and collagen deposition, as demonstrated by H&E staining, Masson’s Trichome staining, and Ashcroft scores assessment (Figure 3B). These results suggest that LTBP2 silencing protects against BLM-induced PF in mice.

Next, we analyzed the inflammatory response of LTBP2 silencing in mice 7 days after BLM treatment. In BLM-challenged mice, a significant increase in the alveolar wall thickness and serious inflammatory cell infiltration were observed. LTBP2 shRNA administration tended to reduce lung inflammation in BLM-treated mice, but this was not significant. Consistently, similar levels of cytokines IL-1β, IL-6, and TNFα were detected in BLM + LTBP2 scr shRNA-treated and BLM + LTBP2 shRNA-treated mice (Supplementary Figure S1E). These data suggest that LTBP2 silencing attenuates BLM-induced lung fibrosis without affecting the inflammatory response in response to lung injury.

### LTBP2 Silencing Inhibits Fibroblast-to-Myofibroblast Differentiation and ECM Accumulation in Fibrotic Lungs of BLM-Stimulated Mice

The differentiation of fibroblasts to α-SMA expressing myofibroblasts is a pivotal event in the process of PF, resulting in excess accumulation of ECM components (Wynn, 2011). Abnormal ECM proteins deposition, including α-SMA, fibronectin, and collagen I, also drives PF progression (Herrera et al., 2018). Therefore, we investigated whether LTBP2 silencing attenuated PF by suppressing fibroblasts’ differentiation to myofibroblasts and decreasing the deposition of ECM proteins.

**FIGURE 4:**
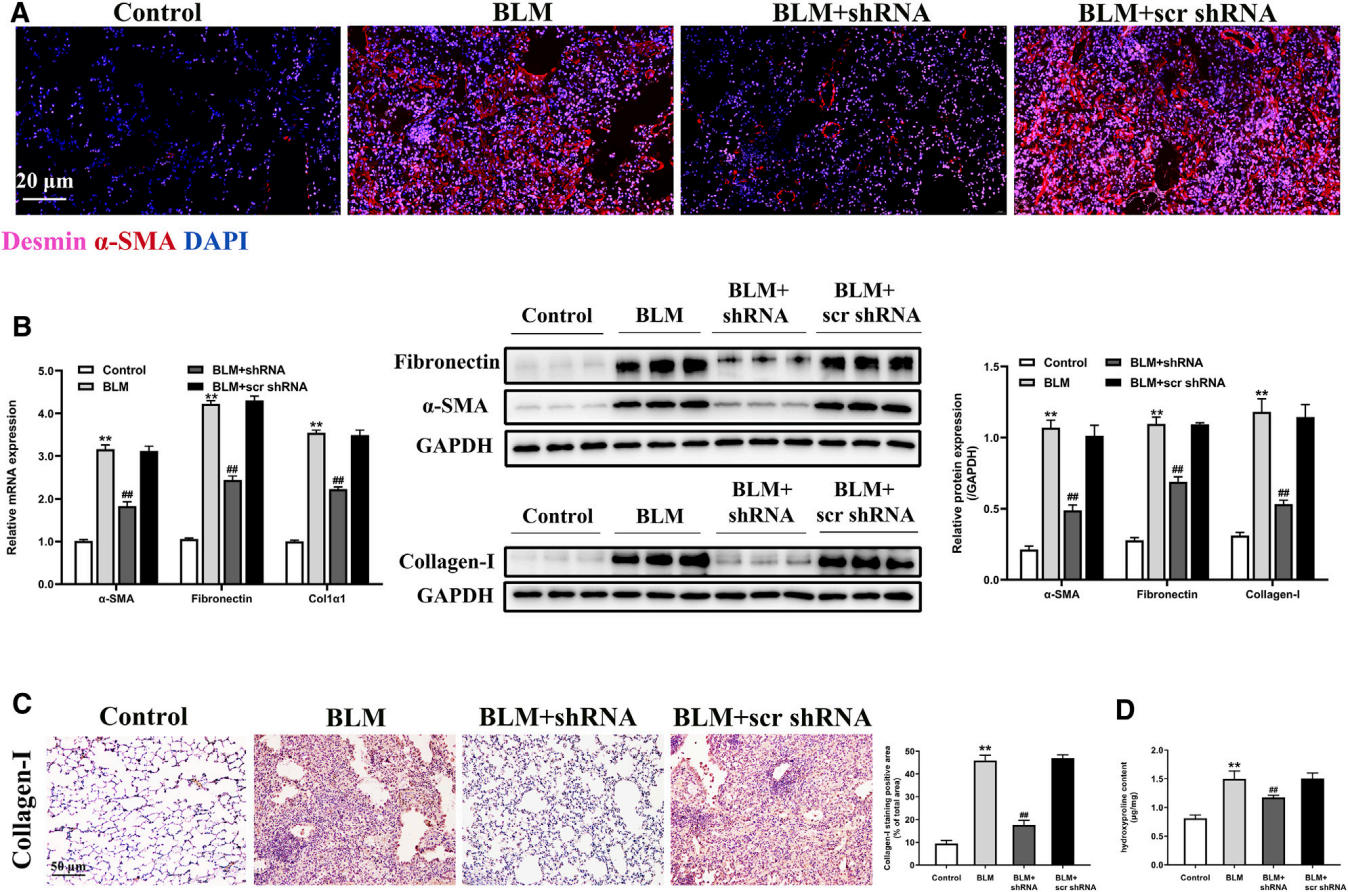
LTBP2 silencing inhibits fibroblast-to-myofibroblast differentiation and ECM accumulation in fibrotic lungs of BLM-stimulated mice. **(A)** Immunofluorescence analysis of desmin (pink) and α-SMA (red) were performed to assess the lung fibroblast-to-myofibroblast differentiation. Nuclei are stained with DAPI (blue). Scale bar = 20 μm. **(B)** Quantitative RT-PCR and western blot analysis of α-SMA, fibronectin, and collagen I in the lungs of mice. GAPDH was detected as the internal control **(C)** Immunohistochemistry for collagen I expression in the lungs of mice. Scale bar = 50 μm. Quantification of positive staining area was measured and statistically analyzed **(D)** The levels of hydroxyproline in the lungs of mice. n = 6, **p < 0.01 vs. control, ^##^ *p* < 0.01 vs. BLM + scr shRNA. Data are represented as means ± SD. Statistical analysis was performed by one-way ANOVA.

Desmin, an intermediate filament protein, can identify stromal cells with fibroblastic morphology. Our results for co-immunostaining suggested that LTBP2 expression was detected in desmin-positive fibroblasts from the BLM + scr shRNA group. However, LTBP2 expression was decreased in those from the BLM + shRNA group (Supplementary Figure S1F). These data suggested that LTBP2 shRNA administration decreased LTBP2 expression in lung fibroblasts. As shown in Figure 4A, desmin-positive lung fibroblasts had a myofibroblast marker (α-SMA) in the BLM-stimulated mice, which verified the involvement of fibroblasts’ differentiation to myofibroblasts in the fibrotic process. Notably, LTBP2 silencing prevented this differentiation. We also found that α-SMA and fibronectin expressions were significantly increased in the BLM-challenged mice. However, LTBP2 silencing significantly blunted upregulation in α-SMA and fibronectin expression levels in mice lungs (Figure 4B). Moreover, LTBP2 silencing significantly reduced lung collagen accumulation, indicated by Western blot, immunohistochemical staining, and the lung hydroxyproline levels (Figures 4B–D). Taken together, these results suggest that LTBP2 silencing is protective against fibrogenesis, possibly *via* limiting the BLM-induced fibroblast-to-myofibroblast differentiation and ECM deposition.

### LTBP2 Silencing Inhibits TGFβ1-Induced Differentiation of Fibroblasts *in Vitro*

TGFβ1 is a key profibrotic cytokine known to mediate the process of PF by lung fibroblasts accumulation and differentiation (Györfi et al., 2018). To determine whether the results in mice were reproducible in human cells, we analyzed the LTBP2 expression in HFL1 cells with or without stimulation by TGFβ1. TGFβ1 increased LTBP2 mRNA level and protein expression in HFL1 cells during fibroblasts’ differentiation to myofibroblasts, which was characterized by the increased expression of the marker gene α-SMA (Supplementary Figure S1G).

**FIGURE 5:**
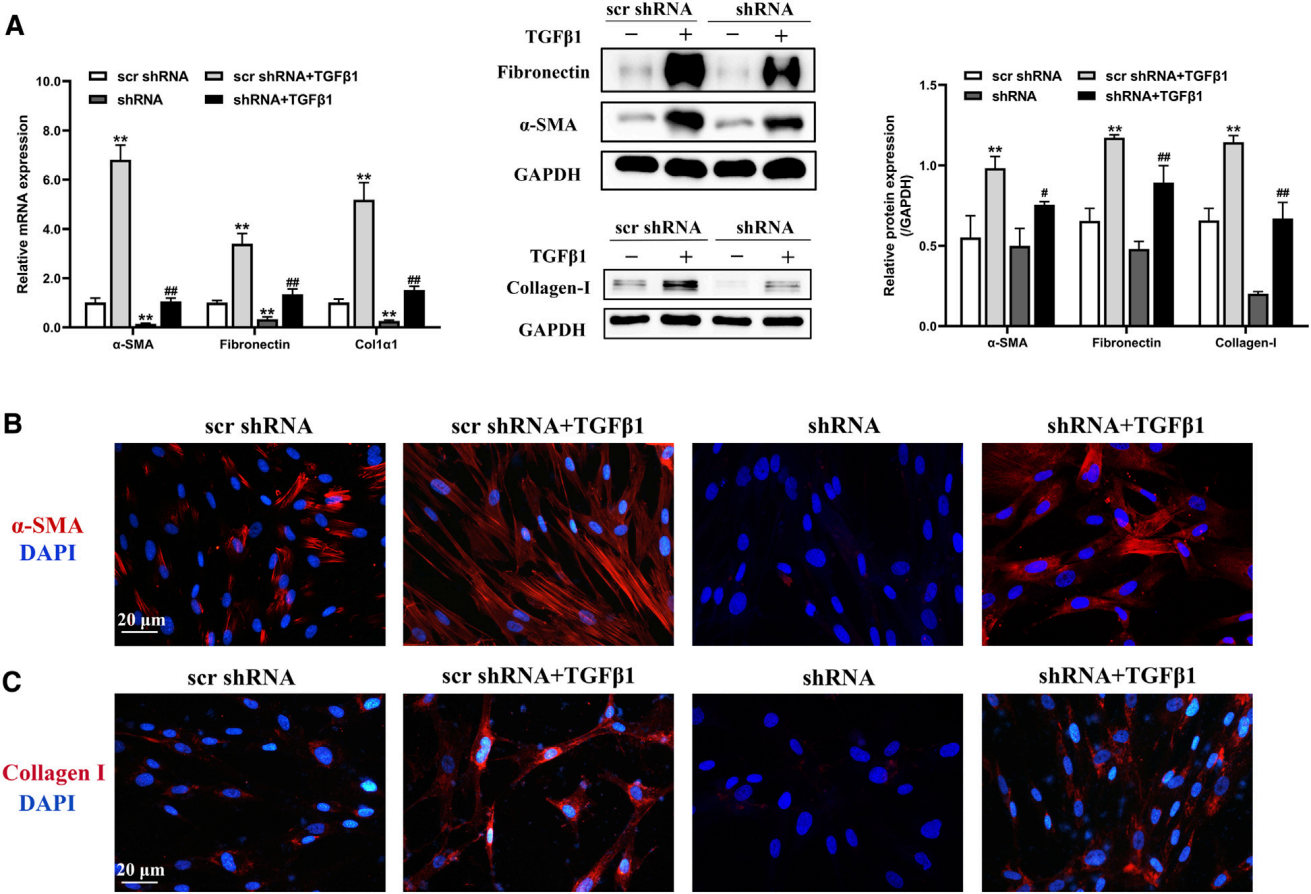
LTBP2 silencing inhibits TGFβ1-induced differentiation of fibroblasts *in vitro*. **(A)** Quantitative RT-PCR and western blot analysis of α-SMA, fibronectin, and collagen I in HFL1 cells transfected with scrambled shRNA or LTBP2 shRNA followed by PBS or TGF-β1 stimulation. GAPDH was detected as the internal control. **p < 0.01 vs. scr shRNA, ^#^ *p* < 0.05, ^##^ *p* < 0.01 vs. scr shRNA + TGFβ1 **(B)** Representative immunofluorescence staining of α-SMA (red) in HFL1 cells. Nuclei are stained with DAPI (blue). Scale bar = 20 μm. **(C)** Representative immunofluorescence staining of collagen I (red) in HFL1 cells. Nuclei are stained with DAPI (blue). Scale bar = 20 μm. Data are represented as means ± SD, n = 3. Statistical analysis was performed by one-way ANOVA.

We next evaluated the effect of LTBP2 silencing on lung fibroblast differentiation by transfecting HFL1 cells with scrambled shRNA or LTBP2 shRNA combined with subsequent TGF-β1 stimulation. We designed three lentivirus shRNAs for human LTBP2 silencing and chose shRNA3 for further experiments because of its silencing efficiency (Supplementary Figure S1H). A significant reduction in the protein expressions of α-SMA, fibronectin, and collagen I was observed in HFL1 cells transfected with LTBP2 shRNA alone compared with scrambled shRNA (Figure 5A and Supplementary Figure S1I). Moreover, LTBP2 silencing abrogated TGFβ1-induced increases in α-SMA, fibronectin, and collagen I in HFL1 cells (Figure 5A). Consistently, immunofluorescence staining showed a lower number of α-SMA-positive cells and less collagen expression in HFL1 cells transfected with LTBP2 shRNA (Figures 5B,C). Collectively, these results suggest that LTBP2 silencing suppresses TGFβ1-induced fibroblasts’ differentiation to myofibroblasts in HFL1 cells.

### LTBP2 Silencing Suppresses the Activation of NF-κB Signaling After BLM Treatment

**FIGURE 6:**
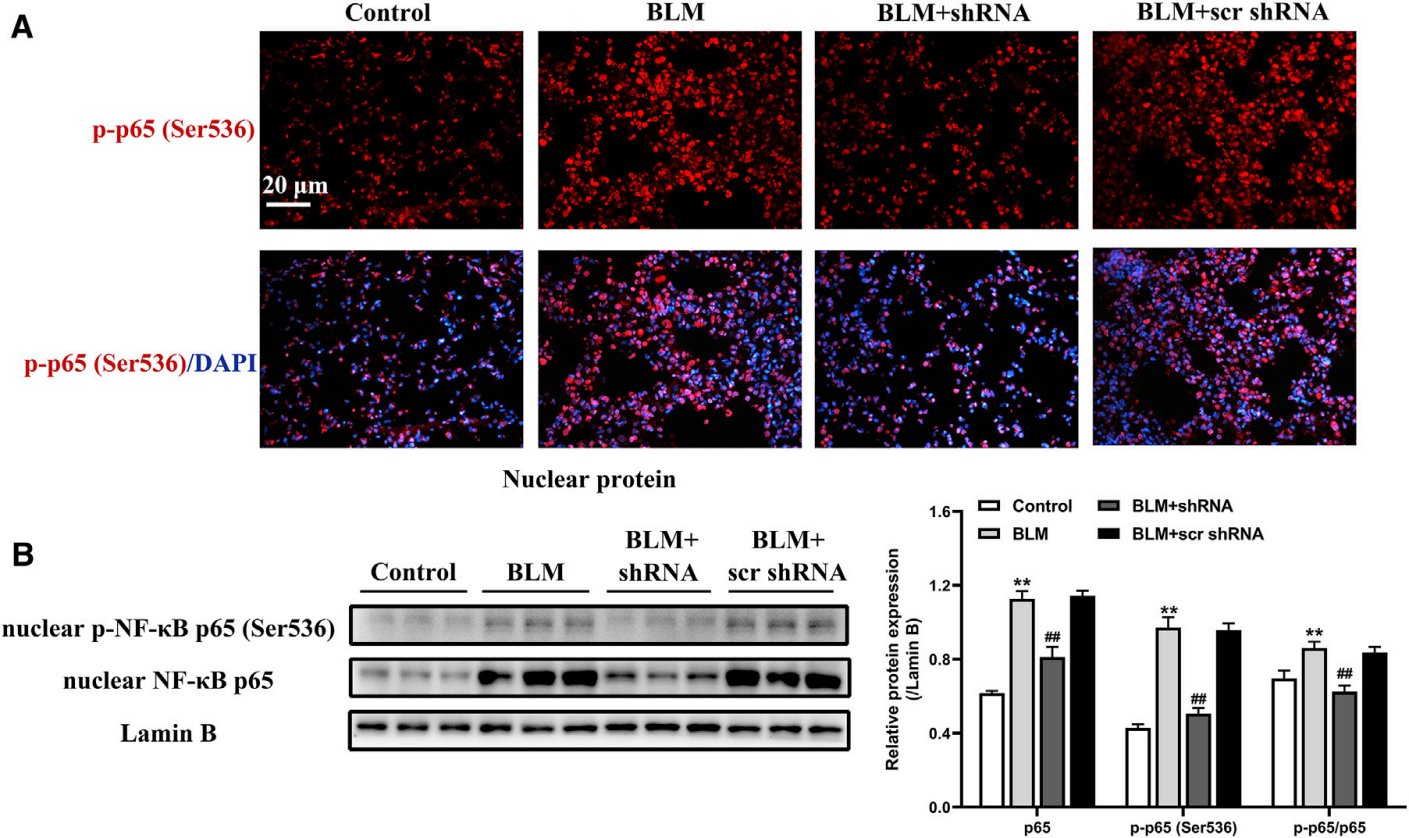
LTBP2 silencing suppresses the activation of NF-κB signaling after BLM treatment. **(A)** Representative immunofluorescence staining of phospho-NF-κB p65 (ser536) (red) in mice lungs. Nuclei are stained with DAPI (blue). Scale bar = 20 μm. **(B)** Western blot analysis of nuclear NF-κB p65 and nuclear phospho-NF-κB p65 (ser536) in the lungs of mice. Lamin B was detected as the internal control. n = 6, **p < 0.01 vs. control, ^##^ *p* < 0.01 vs. BLM + scr shRNA. Data are represented as means ± SD. Statistical analysis was performed by one-way ANOVA.

Increased nuclear translocation of activated NF-κB initiates a cascade of responses, including abundant expression of proinflammatory molecules and profibrogenic cytokines that have been shown to be involved in the pathogenesis of PF (Christman et al., 2000; Meng et al., 2014). To elucidate the underlying mechanism of LTBP2 on lung fibroblasts differentiation and ECM deposition, we analyzed the effect of administering LTBP2 shRNA on NF-κB signaling in mice lungs. The results revealed that BLM administration induced phosphorylated NF‐κB p65 subunit translocation from cytoplasm into nucleus (Figure 6A). Western blot further confirmed that BLM significantly increased both p65 nuclear translocation and phosphorylated p65 levels, confirming the activation of NF-κB signaling (Figure 6B). However, LTBP2 silencing inhibited the BLM-induced phosphorylation and nuclear translocation of the NF‐κB p65 subunit (Figures 6A,B). These results suggest that LTBP2 silencing represses the activation of NF-κB signaling in the BLM-induced lung fibrosis murine model.

### LTBP2 Silencing Suppresses the Activation of NF-κB Signaling Induced by TGFβ1

**FIGURE 7:**
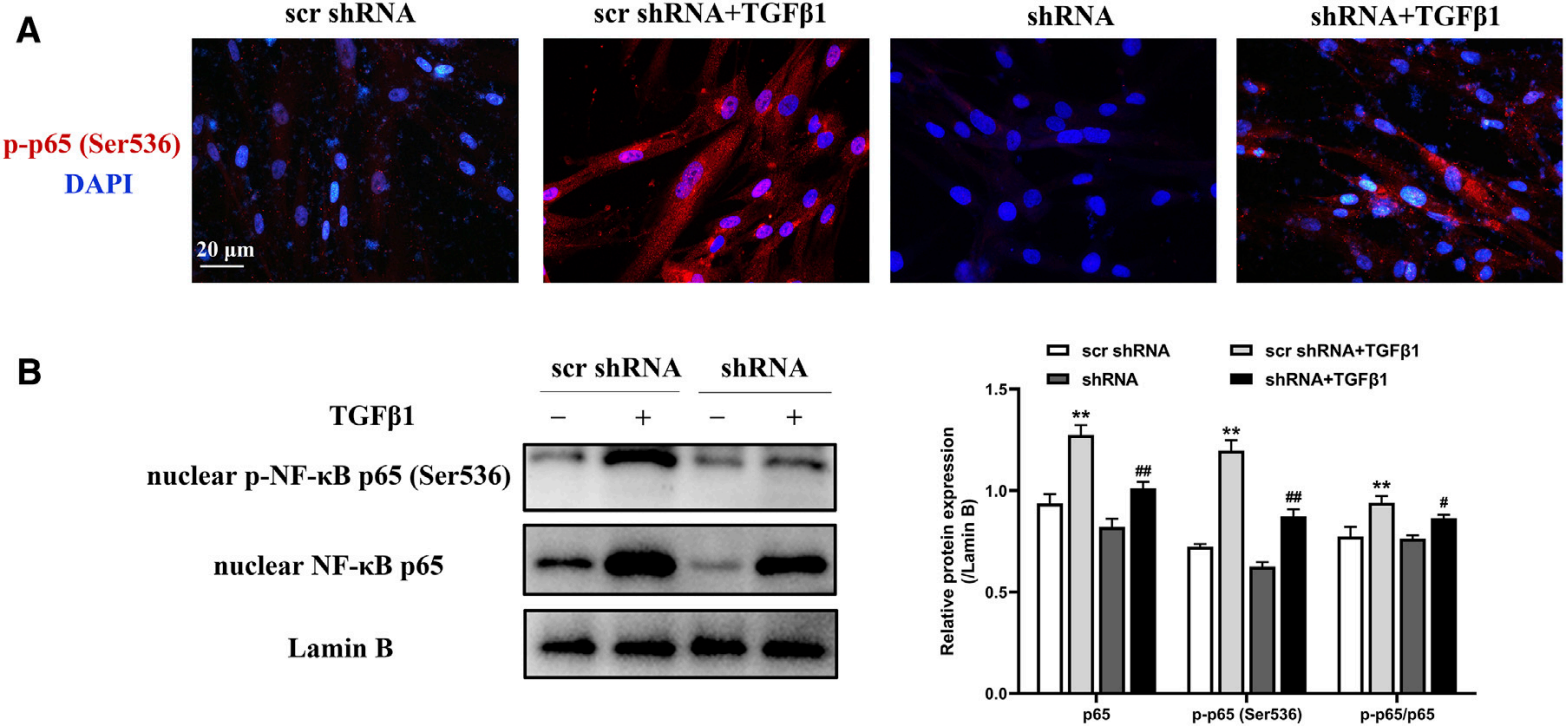
LTBP2 silencing suppresses the activation of NF-κB signaling induced by TGFβ1. **(A)** Representative immunofluorescence staining of phospho-NF-κB p65 (ser536) (red) in HFL1 cells transfected with scrambled shRNA or LTBP2 shRNA followed by PBS or TGF-β1 stimulation. Nuclei are stained with DAPI (blue). Scale bar = 20 μm. **(B)** Western blot analysis of nuclear NF-κB p65 and nuclear phospho-NF-κB p65 (ser536) in HFL1 cells. Lamin B was detected as the internal control. **p < 0.01 vs. scr shRNA, ^#^ *p* < 0.05, ^##^ *p* < 0.01 vs. scr shRNA + TGFβ1. Data are represented as means ± SD, n = 3. Statistical analysis was performed by one-way ANOVA.

As demonstrated previously here, LTBP2 silencing inhibited the phosphorylation and nuclear translocation of NF‐κB signaling in mice lungs. The effect of LTBP2 silencing on TGF-β1 -induced NF‐κB signaling activation in HFL1 cells was subsequently evaluated by immunofluorescence assay and Western blot. As shown in Figure 7, TGFβ1 stimulated phosphorylated NF‐κB p65 subunit translocation from cytoplasm into nucleus. However, TGFβ1-induced phosphorylation and nuclear translocation of NF‐κB p65 subunit in HFL1 cells were blunted by LTBP2 shRNA transfection. These results suggest that LTBP2 silencing suppresses the activation of NF-κB signaling during TGF-β1 stimulation.

### LTBP2 Overexpression-Induced Lung Fibroblast-To-Myofibroblast Differentiation Depends on NF-κB Phosphorylation and Nuclear Translocation *in Vitro*

**FIGURE 8:**
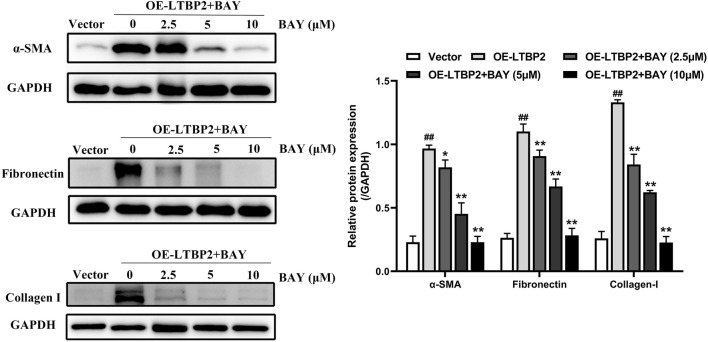
LTBP2 overexpression-induced lung fibroblast-to-myofibroblast differentiation depends on NF-κB phosphorylation and nuclear translocation *in vitro*. Western blot analysis of α-SMA, fibronectin, and collagen I in HFL1 cells transfected with LTBP2 cDNA and/or BAY 11-7082 or vector. GAPDH was detected as the internal control. ^##^ *p* < 0.01 vs. vector. *p < 0.05, **p < 0.01 vs. OE-LTBP2. Data are represented as means ± SD, n = 3. Statistical analysis was performed by one-way ANOVA.

We further examined whether LTBP2 is sufficient alone to regulate lung fibroblast differentiation by conducting a gain-of-function experiment. Interestingly, LTBP2 overexpression induced significant changes in the protein levels of α-SMA, fibronectin, and collagen I (Figure 8 and Supplementary Figure S1J). These results suggest that LTBP2 overexpression is able to induce lung fibroblasts’ differentiation to myofibroblasts in HFL1 cells, even in the absence of TGFβ1.

BAY is a compound in widespread use as an anti-inflammatory agent, and acts as an inhibitor of the activation of the transcription factor NF-κB (Pierce et al., 1997; Hou et al., 2018). Therefore, we next used BAY to investigate the effect of NF-κB signaling on lung fibroblast differentiation induced by LTBP2 overexpression. Our results demonstrated that BAY alone did not affect cell viability, and BAY significantly inhibited LTBP2 overexpression-induced nuclear translocation of the NF-κB p65 subunit (Supplementary Figures S1K, S1L). LTBP2 overexpression induced increases in α-SMA, fibronectin, and collagen I expressions, which was also suppressed by BAY in a dose-dependent manner, with concentrations ranging from 2.5 to 10 μM (Figure 8).

### LTBP2 Expression Is Up-Regulated in RA-ILD Patients and COVID-19-Related PF

**FIGURE 9:**
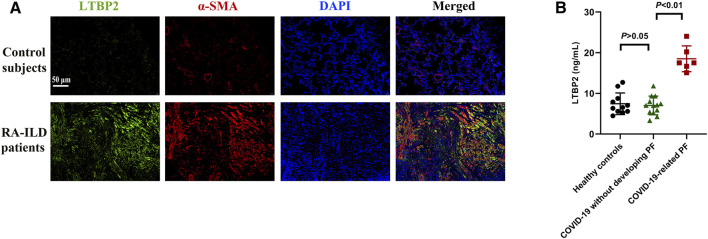
LTBP2 expression is up-regulated in RA-ILD patients and COVID-19-related PF. **(A)** Representative results for co-immunostaining of LTBP2 (green) and α-SMA (red) in the lung sections from patients with RA-ILD (n = 3). Scale bar = 50 μm. **(B)** Serum LTBP2 concentrations in healthy controls (n = 11), COVID-19 patients without PF (n = 13), and patients with PF secondary to COVID-19 (n = 6). Data are represented as means ± SD. Statistical analysis was performed by one-way ANOVA.

We further investigated LTBP2 expression in PF with other different pathological origins. Compared with control group, lung samples originating from RA-ILD patients exhibited increased LTBP2 expression, while co-immunostaining of α-SMA suggested that LTBP2 was co-localized in activated fibroblasts/myofibroblasts (Figure 9A). As LTBP2 was a secreted protein, we measured serum LTBP2 concentrations in patients with PF secondary to COVID-19. Baseline characteristics are summarized in Supplementary Table S3. Serum levels of LTBP2 were similar between healthy controls and COVID-19 patients without PF (mean: 7.45 ng/ml (median: 6.53) compared with 7.04 ng/ml (median: 7.06), *p* > 0.05; Figure 9B). However, serum LTBP2 concentrations in patients with COVID-19-related PF were significantly higher than those in COVID-19 patients without developing PF (mean: 18.51 ng/ml (median: 17.51) compared with 7.04 ng/ml (median: 7.06), *p* < 0.01; Figure 9B).

## Discussion

In the present study, we identified that LTBP2 is upregulated in different types of patients with PF, including IPF, RA-ILD, and COVID-19-related PF, and in mice following BLM-induced PF. Specifically, LTBP2 is overexpressed in activated fibroblasts/myofibroblasts in fibrotic lung tissues. Our data show that LTBP2 silencing protects against BLM-induced PF and suppresses fibroblast-to-myofibroblast differentiation *via* blocking NF-κB signaling *in vivo* and *in vitro*. Interestingly, LTBP2 overexpression promotes lung fibroblast-to-myofibroblast differentiation *in vitro*, even in the absence of TGFβ1, which depends on the activation of NF-κB signaling. Taken together, our results support that strategy aimed at silencing LTBP2 may provide a viable therapy against PF.

LTBP2 has a broad tissue distribution and plays a structural role within elastic fibers in most cases (Shipley et al., 2000). LTBP2 may be associated with organ fibrosis, including the heart (Pang et al., 2020) and skin (Sideek et al., 2016). A previous study showed that serum LTBP2 concentrations in IPF patients were significantly higher than those in healthy controls, but the exact role of LTBP2 in PF had not been well understood (Enomoto et al., 2018). The BLM-induced lung fibrosis model, is able to reproduce many aspects of human IPF and other fibrotic ILDs, and is the best-characterized and most extensively used animal model (Liu et al., 2017). Here, we observed that lung tissues originating from different types of patients with PF, including IPF and RA-ILD, and from mice following BLM-induced PF were characterized by increased LTBP2 expression. Moreover, serum LTBP2 was also elevated in patients with COVID-19-related PF. Consistently, Li et al. (Li et al., 2021) demonstrated that LTBP2 was highly expressed in lungs of mice with PF. More importantly, we found that LTBP2 silencing by lentiviral transfection attenuated BLM-induced PF in mice. Therefore, our results provide evidence of the profibrotic role of LTBP2 in PF.

Fibrotic diseases are typically characterized by a progressive, vicious cycle of abnormally high myofibroblast accumulation. The activated myofibroblast is thought to be the main source of pathological ECM in fibrosis (El Agha et al., 2017; Philp et al., 2018). It has been reported that myofibroblasts in PF could originate from multiple cellular sources, including resident fibroblasts, circulating fibrocytes, epithelial cells, pericytes, and endothelial cells (El Agha et al., 2017; Walsh et al., 2018). Of these, resident fibroblasts make a major contribution to the myofibroblast population in PF (Hoyles et al., 2011) and the differentiation of fibroblasts to myofibroblasts is the primary feature during the progression of PF (Wynn, 2011). Consistent with these findings, we found that fibrotic mice lungs and lung samples originating from IPF and RA-ILD patients exhibited increases in the activated fibroblasts/myofibroblasts marker, and this pathological change was accompanied by increased LTBP2 expression. More importantly, we demonstrated that silencing LTBP2 inhibited fibroblast-to-myofibroblast differentiation and ECM deposition in BLM-treated mice lungs. This is in line with several studies that LTBP2 knockdown decreased collagen expression in myocardial fibrosis (Pang et al., 2020) and ECM gene expression in trabecular meshwork cells (Suri et al., 2018). Moreover, the above *in vivo* findings were further verified by *in vitro* observations that LTBP2 silencing inhibited TGF-β1-mediated fibroblast-to-myofibroblast differentiation in HFL1 cells. Through gain- and loss-of-function assays, we validated LTBP2 was essential and sufficient for TGF-β1-induced lung fibroblast differentiation *in vitro*. It was worth noting that in our study, the differences using the shRNA *in vitro* were less robust than data *in vivo*. We speculated that the results might be related to the difference in the best silencing efficiency of mouse LTBP2 shRNA and human LTBP2 shRNA, and besides fibroblasts, LTBP2 in other types of cells from mouse lungs might play a role in the phenotype through other different mechanisms. Collectively, these findings suggest that LTBP2 is a key regulator of fibroblast differentiation to myofibroblast in PF.

We then further investigated the molecular mechanism by which LTBP2 regulated fibroblast-to-myofibroblast differentiation in PF. As an important transcription factor, NF-κB transcription complexes consists of various homo- and heterodimers, including the subunits p50, p52, c-Rel, RelA (p65), and RelB (Sun, 2017). Previous studies have highlighted the significance of NF-κB signaling in the progression of PF (Inayama et al., 2006; Sun et al., 2015; Hou et al., 2018). Consistent with these reports, we found that the NF-κB signaling was activated by BLM or TGFβ1 *in vivo* or *in vitro*, respectively. Previous data have shown that LTBP2 knockdown inactivated the NF-κB signaling pathway, subsequently decelerating the progression of myocardial fibrosis (Pang et al., 2020). Our results suggested that LTBP2 silencing suppressed the activation of NF-κB signaling *in vivo* and *in vitro*. More importantly, we demonstrated that LTBP2 overexpression directly activated the NF-κB signaling and that LTBP2 overexpression-induced fibroblast-to-myofibroblast differentiation depended on the activation of NF-κB signaling *in vitro*. Contrary to our and others’ results, Kan et al. (Kan et al., 2015) reported that LTBP2 negatively regulated NF-κB p65 by decreasing phosphorylation of p65 at serine 536, inhibiting active phosphorylated p65 nuclear localization and weakening the p65 DNA-binding ability in nasopharyngeal carcinoma. We speculate LTBP2 may have a specific molecular function in each tissue type. NF-κB can be activated by mitogen-activated protein kinases (MAPKs) (Luo et al., 2010). A previous study has already showed that inhibiting MAPKs suppressed the angiotensin II-stimulated NF-κB cascade in HFL1 cells (Meng et al., 2014). Ren et al. (2015) found that upon a transcriptional analysis and interrogation of the KEGG database, LTBP2 knockdown affected the MAPK signaling pathway in HeLa cells. In human MSU-1.1 fibroblasts, a central bioactive region of LTBP-2 stimulated the expression of inactively TGF-β1 *via* p38 MAPK signaling pathways (Sideek et al., 2017). Therefore, in the future, it is necessary to further study the relationship between LTBP2 and MAPKs signaling in PF. While NF-κB is a key signaling pathway targeted by LTBP2 in PF, we cannot rule out the involvement of other signaling pathways which would be very interesting to explore in future studies. In addition, although LTBP2 has not been reported to have transcription factor-like functions, a previous study showed that LTBP2 was one of the super-enhancer-driven pathogenic genes in lung fibrosis and Myc was one of the transcription factors binding to the LTBP2 super-enhancer (Li et al., 2021). Therefore, it’s not ruled out that LTBP2 may directly regulate promoter activity of α-SMA, fibronectin and Col1α, and it would be necessary to explore in the future.

PF is not a single disease. Instead, it is the result of various processes that cause widespread scarring in the lungs. In this study, we investigated LTBP2 expression in three PF with different pathological origins: IPF, RA-ILD and COVID-19-related PF. IPF is defined as UIP based on HRCT and/or histopathological pattern after exclusion of other known causes of ILD (Raghu et al., 2018). IPF patients demonstrate large heterogeneity in their pulmonary manifestation of fibrosis, and it is generally not regarded as an inflammatory disease (Strieter and Mehrad, 2009). RA is a systemic inflammatory autoimmune disorder that can be associated with extra-articular manifestations in up to 50% of patients (Turesson, 2013). Among those with multiple extra-articular manifestations, ILD is the most frequent and worsens the disease prognosis (Bongartz et al., 2010). Contrary to ILD associated with other connective tissue diseases, the most prevalent pattern in RA-ILD is UIP followed by nonspecific interstitial pneumonia (Wu et al., 2019). Compared to RA patients with a non-UIP pattern, a proportion of those with UIP have similar phenotype and mortality as IPF (Song et al., 2013; Samara et al., 2017). The pattern in RA-ILD patients included in the current study is UIP. Moreover, IPF and RA-ILD share some phenotypic characteristics, autoimmune features, and a genetic background (Juge et al., 2020). The hallmarks of IPF and RA-ILD are both fibroblast/myofibroblast hyperproliferation, fibroblast foci formation, and ECM protein deposition. PF has been recognized as a potential sequela among COVID-19 survivors. Virus-induced lung injury, immune response, and attempts at healing are central to the process of fibrogenesis (Ojo et al., 2020). Features of COVID-19 presents some common points with IPF and RA-ILD, typically characterized by a chronic progression over time and possibly complicated by acute exacerbation (Manfredi et al., 2020). Our data showed that LTBP2 expression was up-regulated in patients with IPF, RA-ILD and COVID-19-related PF. However, our study has limitations in BLM-induced PF murine model and it would be necessary to use different animal models to further evaluate the exact role of LTBP2 in RA-ILD and COVID-19-related PF in future studies.

## Conclusion

In summary, our study demonstrates that LTBP2 serves as a pro-fibrotic regulator in pulmonary fibrogenesis and that LTBP2 regulates fibroblasts’ differentiation to myofibroblasts *via* NF-κB signaling. Therefore, LTBP2 may be a potential therapeutic target for the treatment of PF.

## Data Availability

The original contributions presented in the study are included in the article/Supplementary Material, further inquiries can be directed to the corresponding author.

## Supplementary Material

Supplementary Figure S1
**(A)** Gating strategy for sorting of LTBP2-positive and LTBP2-negative murine lung fibroblasts. **(B)** L929 cells were co-transfected with scrambled shRNA, LTBP2 shRNA1, LTBP2 shRNA2, LTBP2 shRNA3. Silencing efficiency was detected by qRT-PCR (*n* = 3, **P* < 0.05, ***P* < 0.01 vs. scr shRNA). **(C)** Mice were received an intratracheal injection of LTBP2 shRNA1-GFP, and mice lung sections were viewed under a fluorescent microscope to assess the transduction efficiency (*n* = 6). Scale bar = 20 μm. **(D)** Western blot analysis of LTBP2 expression in mice lungs. *n* = 3, ***P* < 0.01 vs. control, ^##^ *P* < 0.01 vs. BLM + scr shRNA. **(E)** Mice were treated as indicated and samples were collected on day 7 after intratracheal administration of BLM. Representative Hematoxylin-eosin staining in mice lungs. Scale bar = 100 μm; Total protein concentration in the BALF; The numbers of total cells, macrophages, neutrophils, and lymphocytes counted in the BALF; Quantitative RT-PCR analysis of IL-1β, IL-6, and TNFα in mice lungs. *n* = 6, ***P* < 0.01 vs. control, ^#^ *P* > 0.05 vs. BLM + scr shRNA. **(F)** Immunofluorescence analysis of desmin (red) and LTBP2 (pink) were performed in mice lungs. Nuclei are stained with DAPI (blue). Scale bar = 20 μm, *n* = 3. **(G)** HFL1 cells were treated with TGF-β1 or PBS. Representative immunofluorescence staining, quantitative RT-PCR, and western blot analysis of α-SMA in HFL1 cells. Red, α-SMA; blue, nuclei; Scale bar = 20 μm. *n* = 3, ***P* < 0.01 vs. control; LTBP2 expression in HFL1 cells was assessed using immunofluorescence staining, quantitative RT-PCR, and western blot. Green, LTBP2; blue, nuclei; Scale bar = 20 μm. LTBP2 levels in culture supernatant were measured by ELISA. *n* = 3, ***P* < 0.01 vs. control. **(H)** HFL1 cells were co-transfected with scrambled shRNA, LTBP2 shRNA1, LTBP2 shRNA2, LTBP2 shRNA3. Silencing efficiency was detected by qRT-PCR and western blot (*n* = 3, **P* < 0.05, ***P* < 0.01 vs. scr shRNA). **(I)** Western blot analysis of α-SMA, fibronectin, and collagen I in HFL1 cells transfected with scrambled shRNA or LTBP2 shRNA. *n* = 3, ***P* < 0.01 vs. scr shRNA. **(J)** Western blot analysis of LTBP2 expression in HFL1 cells transfected with LTBP2 cDNA or vector. *n* = 3, ***P* < 0.01 vs. vector. **(K)** HFL1 cells were treated with BAY 11-7082 for 48h. The cell viability was detected by CCK-8 assay kit. *n = 9*, ^#^ *P* > 0.05 vs. control. **(L)** Western blot analysis of nuclear NF-κB p65 expression in HFL1 cells transfected with LTBP2 cDNA and/or BAY 11-7082 or vector. *n* = 3, ^##^ *P* < 0.01 vs. vector. ***P* < 0.01 vs. OE-LTBP2. Data are represented as means ± SD. Statistical analysis was performed by one-way ANOVA or *t*-test.

## Abbreviations

BLM, bleomycin; ECM, extracellular matrix; HRCT, high-resolution computed tomography; IPF, idiopathic pulmonary fibrosis; LTBP2, latent transforming growth factor-β binding protein-2; MAPK, mitogen-activated protein kinase; NF‐κB, nuclear factor kappa B; PF, pulmonary fibrosis; RA-ILD, rheumatoid arthritis-associated interstitial lung disease; α-SMA, α-smooth muscle actin; TGFβ, transforming growth factor-β; UIP, usual interstitial pneumonia.
