# Supplementary figures and images for: Latent Transforming Growth Factor-β Binding Protein-2 Regulates Lung Fibroblast-to-Myofibroblast Differentiation in Pulmonary Fibrosis via NF-κB Signaling
Source: Front Pharmacol. 2021 Dec 24;12:788714. doi: 10.3389/fphar.2021.788714 (PMC8740300; doi:10.3389/fphar.2021.788714)

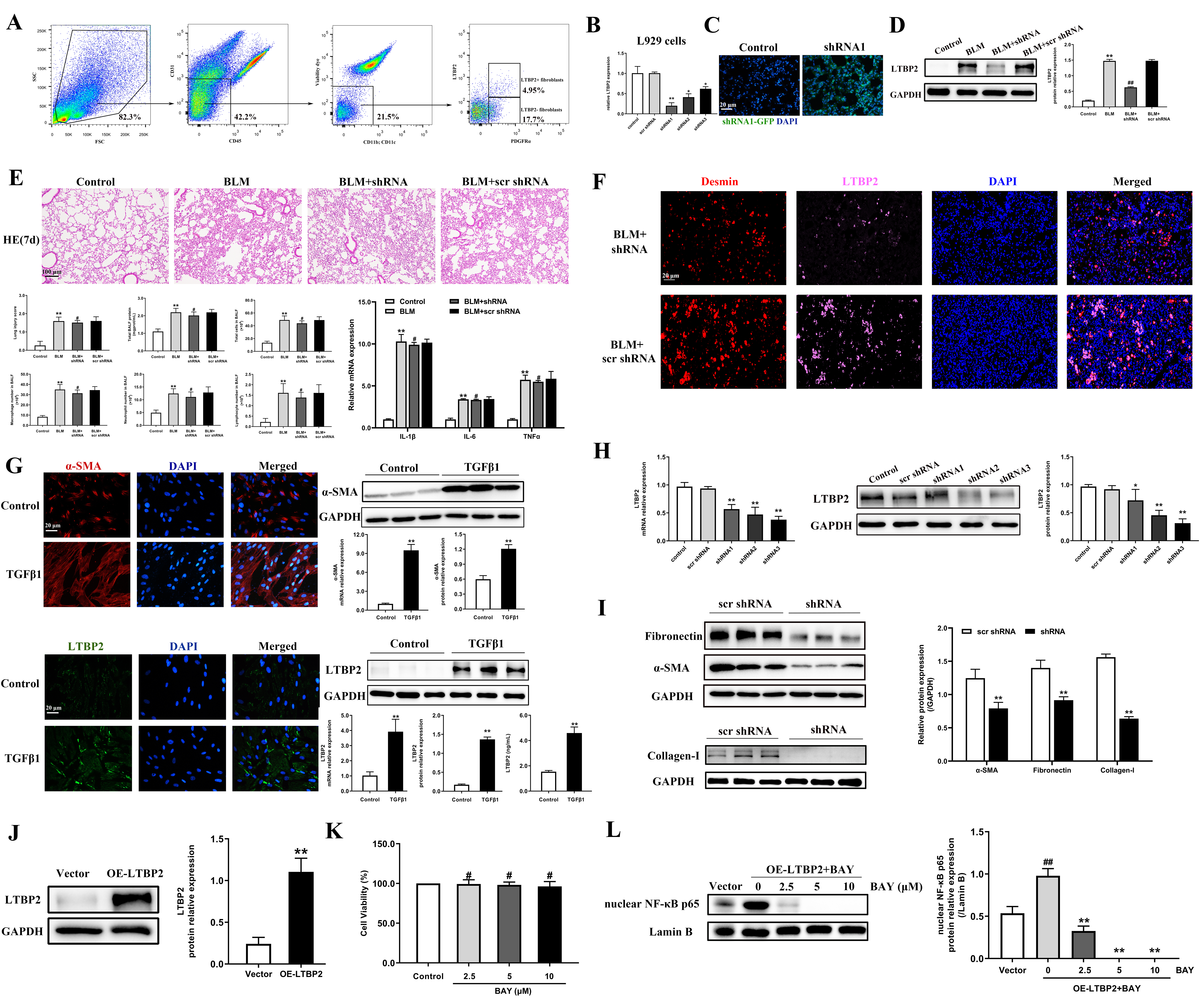

Supplement: Supplementary file 1 [file Image1.jpg]
