# Supplementary material for: Latent Transforming Growth Factor-β Binding Protein-2 Regulates Lung Fibroblast-to-Myofibroblast Differentiation in Pulmonary Fibrosis via NF-κB Signaling
Source: Front Pharmacol. 2021 Dec 24;12:788714. doi: 10.3389/fphar.2021.788714 (PMC8740300; doi:10.3389/fphar.2021.788714)
Supplement: Supplementary file 2 [file DataSheet1.docx]

**Supplementary Table 1.** The target sequences of mouse LTBP2 shRNA

| **ID** | **Target sequence** |
| --- | --- |
| shRNA1 | GCCTCCCAAATGGATACAGAT |
| shRNA2 | CCAGCAGAAGAGCAAGTGATT |
| shRNA3 | CCGTATCTATTTCTGCCAAAT |
| scrambled shRNA | TTCTCCGAACGTGTCACGT |

**Supplementary Table 2.** The target sequences of human LTBP2 shRNA

| **ID** | **Target sequence** |
| --- | --- |
| shRNA1 | CACATGGACATCTGCTGGAAA |
| shRNA2 | GATGCGGATGAGTGTGTGATA |
| shRNA3 | GAAAGGACACTGCCAAGATAT |
| scrambled shRNA | TTCTCCGAACGTGTCACGT |

**Supplementary Table 3.** Baseline characteristics of patients

|  | Healthy controls  n=11 | COVID-19 patients without developing PF  n=13 | COVID-19-related PF  n=6 | *P* |
| --- | --- | --- | --- | --- |
| Age, years, mean ± SD | 57.6 ± 13.5 | 59.2 ± 8.4 | 67.7 ± 13.2 | NS |
| Male | 5(45.5) | 8(61.5) | 4(66.7) | NS |
| Current or former smoker | NA | 6(46.2) | 3(50) | NS |
| Malignant diseases | 0 | 0 | 0 |  |

Data are described as n (%) or mean ± SD. PF, pulmonary fibrosis; NA, not available; NS, not significant.
